# Time Flies—Age Grading of Adult Flies for the Estimation of the Post-Mortem Interval

**DOI:** 10.3390/diagnostics11020152

**Published:** 2021-01-21

**Authors:** Jens Amendt, Valentina Bugelli, Victoria Bernhardt

**Affiliations:** 1 Institute of Legal Medicine, University Hospital Frankfurt/Main, Goethe-University, 60323 Frankfurt/Main, Germany; victoria.bernhardt@hotmail.de; 2 Dipartimento di Scienze della Salute, Sezione di Scienze Medico Forensi, Università di Firenze, 50134 Firenze, Italy; valentina.bugelli@unifi.it

**Keywords:** forensic entomology, pteridine, cuticular hydrocarbons, NIRS, gene expression, proteomics

## Abstract

The estimation of the minimum time since death is one of the main applications of forensic entomology. This can be done by calculating the age of the immature stage of necrophagous flies developing on the corpse, which is confined to approximately 2–4 weeks, depending on temperature and species of the first colonizing wave of flies. Adding the age of the adult flies developed on the dead body could extend this time frame up to several weeks when the body is in a building or closed premise. However, the techniques for accurately estimating the age of adult flies are still in their beginning stages or not sufficiently validated. Here we review the current state of the art of analysing the aging of flies by evaluating the ovarian development, the amount of pteridine in the eyes, the degree of wing damage, the modification of their cuticular hydrocarbon patterns, and the increasing number of growth layers in the cuticula. New approaches, including the use of age specific molecular profiles based on the levels of gene and protein expression and the application of near infrared spectroscopy, are introduced, and the forensic relevance of these methods is discussed.

## 1. Introduction

The age estimation of necrophagous blow fly larvae and pupae as a function of ambient temperature and species is the key task of forensic entomology when it comes to the identification of the minimum post-mortem interval (PMI_min_), which is the time between the first insect colonization of a body and its discovery [1,2]. As adult blow flies are able to colonize a corpse within minutes after death, age estimation of their juvenile stages may yield “on-the-day” data for the PMI_min_ even several weeks post mortem. They are therefore the most important diagnostic tool in the scientific estimation of time since death in forensic medicine. An even greater extension of this period “several weeks post-mortem” would be nevertheless a great success.

**Figure 1:**
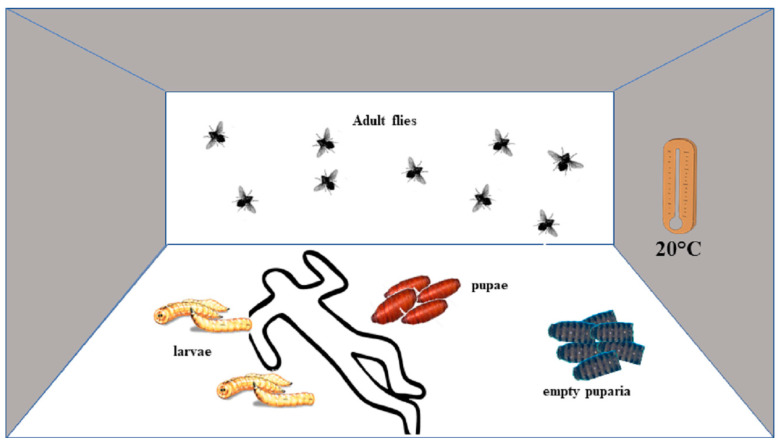
An exemplary PMI_min_ determination using a fictitious case. The dead body was discovered in an apartment (20 °C ambient temperature), and only larvae, pupae, and empty puparia of one single species, the blow fly *Calliphora vicina*, were found on the body and its surroundings. In order to determine the PMI_min_, the most advanced stage of its development will be of relevance. In the present case, these are empty puparia. Since there is no current method to narrow this down with respect to the time of hatching, the entomologist will assume that the flies hatched immediately before the body was found in order to determine a reliable PMI_min_. There is a risk that this may lead to an underestimation of the time period. According to, e.g., Marchenko [11], *C. vicina* needs about 21 days to complete a complete development cycle at a temperature of 20 °C. These 21 days would be the PMI_min_ in the present case, based just on the immature specimens (larvae, pupae) and remains (empty puparia). However, there are hundreds of adult *C. vicina* in the apartment. Thirty to forty specimens are captured, and by using various techniques (see below), their age is estimated to be 15–17 days. Based on the assumption that the animals have developed on the cadaver, this now means that we are giving a PMI_min_ of 36 days in the entomological report (21 days development of larva and pupa plus 15 days aging of the fly).

Quantifying the weathering and chemical degradation of empty fly puparia (the shells, in which the transformation of the maggot via the pupal stage into the adult fly takes place) discovered at a scene of a crime could be one opportunity, but relies on Gas Chromatography—Mass Spectrometry methods and equipment, and is still tentative, as the weathering varies among species. Moreover, puparia might be overlooked due to the tendency of maggots to seek sheltered places to pupate and therefore not be collected by an untrained investigator. Last but not least, a variety of different methods should always be considered in order to minimize the problem of variability and natural noise of biological systems or living animals. Just as different methods of age determination of juvenile necrophagous insects (further breeding to the adult stage, length measurements of larvae, etc.) improve the accuracy of forensic entomological expertise, different techniques can also usefully complement each other in the analysis of the remains of the necrophagous fauna. Adult flies at the scene offer an important complement and support here [3,4,5]. Senescence or aging is the progressive deterioration in the physiological state of an organism over time that culminates in death [6,7]. In the adult fly, it is linked with various morphological and molecular variations, and by using these variations for the aging specimens, which remain on the scene of the body after finishing their development, could open a new door to the estimation of the PMI_min_. This applies especially to indoor scenes, where the majority of routine cases of insect infested bodies are located [8], and where hatched flies have difficulty leaving the room due to closing conditions. While under field conditions, a distinction between flies just arriving and those which developed on the body is difficult to achieve, at indoor scenes many of the flies originating from the cadaver may be still present at the scene for several weeks [9], and demonstrated that it is possible to establish the origin of a population of such flies at a crime scene by means of a morphology examination. As adult flies might reach ages of up to 68 days [10], adding their age to the time required for larval development and metamorphosis could extend the calculable period by several weeks (Figure 1). The present review summarizes the current state of the art of estimating the age of adult flies and evaluates possible problems and pitfalls regarding techniques and case work.

## 2. Age-Grading Targets

### 2.1. Morphology

#### 2.1.1. Reproductive System

Examining qualitative or quantitative changes in the reproductive morphology of an insect is one of the oldest methods of age grading. Morphological changes in the reproductive system of maturing flies are well studied and have been described in several reviews [12,13,14]. Only few studies demonstrated age dependent changes in the male reproductive system. Ponlawat and Harrington [15] showed, e.g., a positive correlation of the number of spermatozoa with age, which also correlates with body size in *Aedes aegypti* (Diptera: Culicidae). Mahmood and Reisen [16] found that the number of total and mature spermatocysts in the mosquito *Anopheles culicifacies* (Diptera: Culicidae) declined significantly with age, but also noted that the changes in males may be reversible after a rest period and are therefore misleading. Studies on age dependent changes in males of forensically relevant Diptera like blow flies are still missing. Instead, age estimation of various Diptera species focuses on the female ovarian development [17,18,19,20,21,22,23,24] e.g., by evaluating the stages of egg development [19] or the examination of ovarioles for follicular relicts to determine the number of ovarian cycles [25]. Krafsur et al. [26] applied age-grading techniques based on ovarian morphology of the muscid stable fly *Stomoxys calcitrans* and Sutherland [27] defining age categories to distinguish between newly-emerged, nulliparous and uniparous females of *S. calcitrans* and females that have reproduced twice (biparous) or more (pauciparous). This kind of staging is also established for forensically relevant Muscidae or blow flies like *Chrysomya* spp. [28,29,30,31] The dependence on protein sources for egg formation and the absence of oviposition sites, which could delay the egg formation, but also the numbers of ovarian cycles in female flies, are a disadvantage of these methods, as they are the source of significant variation [12]. Moreover, Gillies and Wilkes [32] highlighted the lack of reproducibility of this technique across species and between technicians during their work on *Anopheles* spp.

#### 2.1.2. Cuticle Growth Layers

Cuticle growth layers are on the internal cuticular projections of the exoskeleton on which muscles attach and are the result of the cuticle deposition rhythm, which is under control of circadian clocks in epidermal cells, as shown for *Drosophila melanogaster* [33,34]. After eclosion, epidermal cells secrete additional material in order to thicken the endocuticle, which is, beside epi- and exocuticle, part of the exoskeleton. Hence, counting those regularly growing cuticle layers seems to be an appropriate age estimation tool, as they are comparable with annual rings of trees, but occur here on a daily basis [35]. Schlein and Gratz [36,37] demonstrated a daily cuticle growth at two thoracic structures for various Diptera species like *Sarcophaga falculata*, *Calliphora erythrocephala (=vicina)*, *Glossina austeni*, *Culex pipiens molestus*, *Aedes aegypti*, and *Anopheles gambiae.* Johnston and Ellison [38] observed similar growth layers at one of these structures in different strains of *Drosophila* sp., and Tyndale-Biscoe and Kitching [39] used cuticular growth rings to determine age in field studies on the sheep blowfly, *Lucilia cuprina.* They are visible in a light microscope and, according to [40], it was found that for the screwworm *Cochliomyia hominivorax*, agreement with known age was within plus or minus one day. However, the latter study was performed only on laboratory-reared specimens and differentiated between two age cohorts (6 versus 15 days). A detailed overview on this topic regarding various insect families and species is given by Neville [41,42]. Even if the great simplicity of the method at first sight is convincing, one should keep in mind that there is a certain abiotic bias (e.g., light-dark regime and temperature adjustments), moreover the classification/identification of the different cuticle layers is not a simple routine task. Last but not least, these growth bands might be visible until a certain age only.

#### 2.1.3. Wing Fray

Insects accumulate small wing injuries (so called wing frays) [43] during their life time, which can be used for evaluating the age of the specimen [44,45]. This method was already applied in a variety of fly families like Glossinidae [46], Scatophagidae [47], or Psychomyiidae [48]. Hayes et al. [44] adapted this method for forensically relevant blow flies. The authors developed a “wing fray index”, which comprises the distance of missing parts of wing margins and of the length of the posterior cross vein. Davies [49] published a study on *Calliphora vicina* where he defined five classes or states of wing fray based mainly on the number of indents on the fringe of the wings and the percentage of its damage. Beside the potential benefit of this technique, there are serious shortcomings, as the amount of wing fray is not solely linked to the age of the fly, but to its life history in terms of activity and wing movement, predator attack, and intraspecific interactions [44]. This is the reason why Beutler et al. [9] recommend the use of wing fray mainly to determine if flies sampled at an actual crime scene had developed on the body or are wild flies attracted to the body (showing a high degree of wing fray), but not as a sound tool for estimating the age of a fly.

### 2.2. Biochemistry

The numerous metabolic processes in flies leave traces that can sometimes be tracked and used as time markers.

#### 2.2.1. Pteridines

Pteridines derive from a pyrimidine-pyrazine ring and are 2-amino-4-hydroxy derivatives [50,51]. They had been first identified by Hopkins in the wings of pierid butterflies in 1895 [52]. Together with ommochromes, pteridines represent the pigments in the ommatidia of the insect compound eye [50,51] where they are either coloured or colourless. Their stability allows for their long-term accumulation in the eyes of Diptera. The so called drosopterins contribute to the red [53], and sepiapterin and isosepiapterin to the yellow-brown pigmentation [51]. Some pteridines have been considered to be just degradation products [51], but some authors suggested that they may function as a light filter in the near UV and blue regions [51,54]. In 1965, Patat [54] studied pteridine patterns in the eyes of the blow fly *Calliphora erythrocephala* (=*vicina*) and observed that pteridines showed age-dependent quantitative changes in their composition. From eclosion of the adult flies to Day 10, the amount of pteridines increased linearly and thereafter showed constant levels. Summers and Howells [55] demonstrated that in wild type and eye colour mutants of another blow fly, *Lucilia cuprina*, the pteridine sepiapterin increases shortly before eclosion of the fly and steadily increased thereafter.

In a study by Mail et al. [56], pteridine accumulation was firstly described as an age-grading parameter for the adult stages of the blood feeding *Stomoxys calcitrans* (Diptera: Muscidae). In this study, pteridines were shown to increase consistently in a linear manner and did not exhibit constant levels at a certain point as described by Patat [54] and Summers and Howells [55] for blow fly species. For a number of dipteran species, pteridine amounts were measured as a function of age, temperature, and sex. While an age dependent, (curvi-) linear increase was demonstrated for a broad range of Diptera, some exceptions exist, such as for the Culicidae species [57,58]. Recently, Bernhardt et al. [59] and Dimitrov et al. [60] presented for both sexes of the two forensically most important blow flies of central Europe, *L. sericata* and *C. vicina*, key reference values for the age-related accumulation of pteridine at room temperature. Cammack et al. [61] analysed pteridine accumulation for the forensically relevant blow flies *Chrysomya megacephala*, *Cochliomyia macellaria*, and *Phormia regina*, when reared at temperatures ranging from 5 to 35 °C. Age could be estimated for almost all temperature–sex combinations, underlining the promising usability of this method.

Due to the curvilinear increase in some species, an age-estimation may not be applicable for the complete adult stage. Moreover, Křemenová et al. [62] showed for several bed bug species that the accumulation of particular pteridines varied between different populations and rearing temperatures. As pteridines have unique absorption spectra, there is the potential for a fast and low-cost quantification by spectrofluorometry [59,60,61]. However, simple fluorimeters have a low laboratory specific sensitivity and may not be able to withstand testing or validation under field conditions. More sophisticated methods like high-pressure liquid chromatography may be better suited to provide reproducible results even under environmental = natural conditions. Moreover, the authors stated that more than one standard should be applied and measured simultaneously to subsequently select those that show consistent changes over time [62]. In the end, however, the effort should pay off, as such a decrease could be observed over a very long period of time: Ref. [63] proved that the ant *Platythyrea punctata* shows a decrease in pteridine levels over time until 70–80 days of age, and if such long lasting aging processes could also be elucidated in detail in flies, this would be a great benefit in FE.

#### 2.2.2. Cuticular Hydrocarbons

In insects, a thin lipid wax layer mainly composed of hydrocarbons, alcohols, waxes, acylglycerides, phospholipids, and glycolipids covers the cuticle [64,65,66,67]. Insect cuticular hydrocarbons (CHC) often consist of complex mixtures of straight chain, unsaturated, and methyl-branched components with up to 40+ carbons. They function to restrict water loss and desiccation, and they facilitate chemical communication in many species [64,68,69,70,71,72]. In social insects, this layer, e.g., functions in nestmate recognition, task decision control, or caste recognition [72]. It is secreted by oenocytes, which are of ectodermal origin and vary in size, number, and anatomical location among species [73].

Jackson et al. [67] compared the CHC composition of newly eclosed, 7-days-old, and 19-days-old adults of the flesh fly *Sarcophaga bullata.* They showed that the total amount of CHC’s increased with age, especially CHC’s with a chain length between C25 and C32. Uebel et al. [74,75,76,77] also described age-dependent changes in the cuticular CHC composition for *Musca autumnalis*, *Fannia canicularis*, *Fannia pusio*, and *Fannia femoralis*. In their study on *Drosophila virilis* (Diptera: Drosophilidae), Jackson and Bartelt [78] showed that the quantity of total CHC’s increased 3-fold between Day 0 and Day 4 in both sexes, but after Day 4 the quantity increased 2-fold in male flies, while it remained unchanged in females until Day 8. The authors also observed that the average chain length of CHC’s decreased with increasing age [78]. In the following years, the first blow flies of forensic interest were investigated regarding age-dependent changes of their CHC composition, but these findings were not linked with PMI_min_ estimations [79,80,81,82,83]. In 2008, Roux et al. [84] described CHC assays as potential age-grading tools for juvenile and adult stages of the forensically relevant blow flies *Protophormia terraenovae*, *Calliphora vomitoria*, and *C. vicina*. Later on, only a few studies on CHC’s as age-grading tools of adult blow flies like *C. macellaria*, *Chrysomya rufifacies*, and *Chrysomya putoria* have been published [66,85]. However, their relevance in forensic entomological casework is low, since age-dependent changes are, at least partly, minor, and clear CHC profiles on a daily basis have not been presented yet. Bernhardt et al. [86] analysed the CHC n-pentacosane (nC25) on the legs of the adult blow flies *L. sericata* and *C. vicina* with gas chromatography–mass spectrometry at room temperature until day 20 post-emergence. The amounts of nC25 increased linearly in both species, but revealed differences between the two sexes in *L. sericata*. The authors provide sex-specific equations for both species for the prediction of fly age. There are indications for abiotic effects, such as diet, temperature, humidity, and laboratory-rearing-effects [64,87,88], which may have an impact on the CHC abundance. Despite that, CHC-based age-prediction models for mosquitos have been validated against field caught specimens and seem robust up to about 15 days [89].

One of the most recent innovations for differentiating a variety of insect traits (age, species, and infection) based on chemical compounds has been the use of near-infrared spectroscopy (NIRS) [90]. NIRS measures the absorption of organic compounds within a sample using an electromagnetic spectrum in the near-infrared region (350–2500 nm) [91] and subsequent analysis may detect compositional differences between samples according to the near-infrared energy absorbed [90]. It is difficult to attribute specific signals to particular chemical signatures, but it seems that age-related degradation of, e.g., surface CHC’s on insect exoskeletons creates unique absorption spectra [41,92]. This is the proposed basis of the capacity of NIRS-based models to place laboratory-reared, adult *An. gambiae* s.s. mosquitoes into binary age categories (e.g., less than and greater than 7 days old) with a reported accuracy of approximately 80% [93,94,95]. NIRS has been described as an age-grading technique by Perez-Mendoza et al. [92] in studies on *Musca domestica*, *Stomoxys calcitrans*, and *Musca autumnalis*. They used whole flies, fresh and dried heads, and identified age-dependent changes in -CH3, -CH2, and -CH functional groups of not further described molecules. However, the authors stated that their calibration model for regression equations is not transferable, since they are unique for each instrument. Villet and Amendt [96] suggested age estimation of adult necrophagous fly species with NIRS, but unfortunately no forensic motivated studies were performed until today. Voss et al. [97] published a study on reflection profiles of blow fly pupae, including infrared wavelengths, for the age and species identification. Recently, developments around NIRS technology have improved [98,99] and moreover expanded to related methods using the midinfrared region (MIRS; 2500–25,000 nm) for spectral analysis [100]. This wavelength penetrates less deeply into the target than NIRS, and is easier to handle in terms of the biochemical differences identified. Spectral outputs are also less sensitive to the moisture content of the sample. Early evidence for yellow fever transmitting *Aedes aegypti*, derived from 2- and 10-day-old, laboratory-reared specimens, showed that these can be separated with 0–3% prediction errors [100,101].

### 2.3. Genetics and Proteomics

#### 2.3.1. Genes

Gene expression can be measured by using reverse transcriptase quantitative PCR (RT-qPCR) and by this, correlation between the transcription products of multiple genes and age-dependent expression patterns can be examined. Gene expression analysis as an age-grading tool for forensically important blow flies so far is exclusively performed on immature stages by studying changes in mRNA levels during development because genes are temporally up- and down-regulated to achieve appropriate progress in development [102,103,104,105]. First data of gene expression patterns for adult stages of Diptera were obtained by studies on *D. melanogaster* [106,107,108,109] and on *Ae. aegypti* [110]. Cook et al. [110,111] identified orthologous *D. melanogaster* genes in *Ae. aegypti* and Marinotti et al. [112] published a genome-wide analysis of gene expression in the adult stages of *Anopheles gambiae* and detected age related expression patterns, which depended on blood meals. Extensive genomics work on *Drosophila* revealed several more candidate genes, which may be worth analysing in blow flies and in other forensically relevant taxa as well. One possible target is the genetic background of the neurotransmitter dopamine, which is known to be involved in a multitude of physiological processes. Bednářová et al. [113] showed recently that old *D. melanogaster* specimens exhibited different levels of dopamine, which additionally are sex-dependent. Already DeLuca et al. [114] mentioned that genes which are important for the synthesis of dopamine producing enzymes (like *Ddc*) are potential candidates for life span analysis, and Carnes et al. [115] found significant differences in gene expression with the population and the age for *D. melanogaster* populations. However, the method requires extensive target identification and optimization and from a forensic perspective the technological effort, the costs, and the need for species-specific profiling do not make it seem realistic that this method will prevail in routine applications. Recently, Martinson [116] provided groundwork by establishing sex and developmental-specific gene sets for the flesh fly *S. bullata*, based on RNA-sequencing. This work can be seen in one line with various other recent transcriptomic and genomics studies focusing on carrion-feeding flies of the forensically relevant blow fly genera *Lucilia* and *Chrysomya* [117,118,119,120]. However, despite the fact that there is a growing body of literature, there are no applicable data sets for estimating the age of an adult blow fly due to gene expression and/or RNA data.

#### 2.3.2. Proteins

Age-dependent changes to the expression of proteins also provide biomarkers for age grading. An earlier study by Fleming et al. [121] analysed quantitative protein expression patterns during the life-span of *D. melanogaster* by means of two-dimensional protein gel patterns at “young”, “middle”, and “old” flies and found significant age-related quantitative changes. Much research is done on mosquitos, as their age is a crucial determinant of their ability to transmit pathogens and their resistance to insecticides. Hugo et al. [122] tested proteomic biomarkers as possible age-grading tools in *Ae. aegypti* and could detect four promising candidate proteins like, e.g., actin depolymerising factor, which decreased in adultsbetween Day 1 and Day 34 after eclosion. Similar results have been presented by Iovinella et al. [123] for *Aedes albopictus* and by Chang [124] for *Bactrocera dorsalis.* Sikulu et al. [125] identified several proteins with characteristic changes in abundance in both *A. gambiae* and *A. stephensi* during their aging process. Rabani et al. [126] recently published a study on two forensically relevant taxa but in a non-forensic context. Moreover, they were just examining larval stages. No studies on adult specimens of forensically relevant flies exist which investigate changes on protein expression level in an age-related manner.

## 3. Conclusions

The age determination of adult flies could extend the day-exact identification of a PMI_min_ period by several weeks, but many of the current methods of determining their age seem not yet robust enough to meet forensic requirements. This can be seen in the possibilities of the promising genetic methods: Tomberlin et al. [127] suggested that quantitative genetic studies may contribute to understand variation in phenotypes and to what extent phenotypes are affected by individual genetic differences and by their interaction with the environment. These interactions of genotype, phenotype, and environment are one of the most exciting, but also difficult, challenges in the near future when it comes to age grading of adult flies in a forensic context.

**Figure 2:**
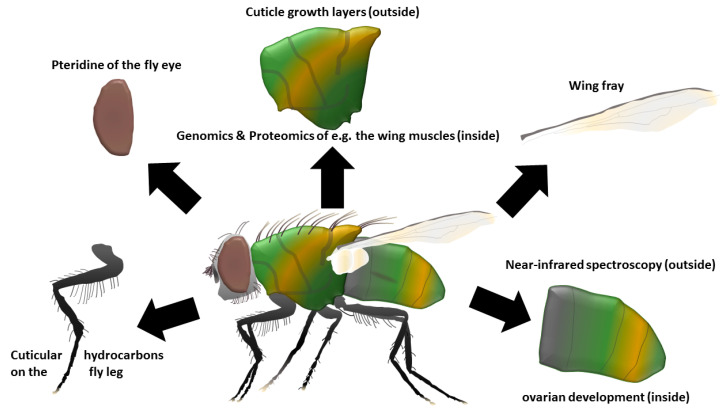
Different methods can use/analyse one and the same adult specimen by different techniques.

The application of a single method for age estimation of an adult fly seems to be moderately successful and may provide a rough idea of a specimen’s age, but not on a daily basis. Reliable age estimation of adult flies might be achieved in the future by the simultaneous application of several methods, as shown by Wall et al. [23], Perez-Mendoza et al. [92], Moon and Krafsur [128], or Butler et al. [129]. Pteridine, CHC assays, and the evaluating of cuticular bands and ovarian development are currently most promising (and mainly inexpensive) (Table 1) and may be performed with the head, legs, wings, thorax, and abdomen of just one and the same specimen. Being able to use the same sample for many different methods is one of the great advantages of age determination of adult flies (Figure 2), together with the rather easy sampling and storage of the evidence: Dry storage at room temperature (if possible, in the dark) seems to be recommended for many of the methods, at least if a timely transfer is ensured. After receiving the samples, the material can be examined morphologically and biochemically without much preparation and provide initial results within 24 h.

The question that remains to be answered is whether the flies studied are actually the F1 generation of the population developing on the corpse. If the adults have little wing damage and a high proportion of males, this would indicate an F1 or later generation of adults, i.e., that they developed on the body [9]. If there are mostly female with a significant amount of wing damage, investigators could conclude that these flies came from the outside. The flies’ nutritional history might also reveal whether they developed on the scene. Bernhardt et al. [130] compared the isotope signatures (here: The stable carbon (δ^13^C) and nitrogen (δ^15^N)) of tissue from humans and 12 additional vertebrates, and from the flies developing on these tissues of the adult flies. They concluded that the fly patterns mirrored the isotope signatures for the respective tissues on which they developed as larvae. The authors recommend that the isotope signatures for the body in question should also be determined so that they can be compared with the signatures from the entomological specimens from the crime scene.

Last but not least, an important extension of the optional analysis of adult flies is the clarification of the extent to which the different methods or targets also apply to dead specimens. As there may not be a source of water or sugar to supply emerging flies, and they frequently fly to a window where they dehydrate quickly, the sampling of adult flies at a scene will most likely be of dead specimens. Here, proteomics and genomics are not promising tools, but, e.g., pteridines and NIRS could be still useful.

## Figures and Tables

**Table 1:** Summary of existing methods [in brackets key references] and an assessment of their current value/benefit with a scale from 1: bad to 5: excellent.

Technique		Pro	Contra	Value
	Reproductive system [25,27,31]	easy to do preparation	mainly just for females rough classification into categories	3
Morphology	Cuticle growth layers [36,38,39]	easy to do preparation, day-precise information	Identification and categorisation of different cuticles not a simple routine task	2
	Wing fray [9,43,46]	easy to classify	not solely linked to the age of the fly but to its life history rough classification into categories	1
Biochemistry	Pteridines [59,61,62]	easy to do extraction many different pteridines = targets some analytical techniques are low cost	Species and sex specificity makes it necessary to produce separate references for each taxa	4
	Cuticular hydrocarbons [72,84,92]	easy to do extraction	Sophisticated analysis and evaluation Species and sex specificity makes it necessary to produce separate references for each taxa	4
Genetics & proteomics	Genes [111,112,116]	Easy to do extraction and applying a simple method (RT-qPCR)	requires extensive target identification and optimization, i.e., expensive laboratory work Species and sex specificity makes it necessary to produce separate references for each taxa	2
	Proteins [111,112,116]	easy to do extraction	No data at all for species of forensic relevance expensive laboratory work	1
